# “Development and validation towards a Nomogram to predict acute kidney Injury following PCNL”

**DOI:** 10.1007/s00345-025-05511-w

**Published:** 2025-02-24

**Authors:** Abhishek Goli, Kasi Viswanath Gali, Arun Chawla, Sunil Pillai Bhaskara, Padmaraj Hegde, Ankit Agarwal, Email Id, Jean de la Rosette, Pilar Laguna, Bhaskar Somani

**Affiliations:** 1 Department of Urology and Renal Transplant, Kasturba Medical College, Manipal, India; 2 Department of Urology and Renal Transplant, Kasturba Medical College, Manipal Academy of Higher Education, Manipal, 576104 Karnataka India; 3 Department of Urology, Istanbul Medipol University, Istanbul, Turkey; 4 Department of Urology, University Hospital Southampton, Southampton, UK

**Keywords:** Percutaneous nephrolithotomy, Acute kidney injury, Risk factors, Nomogram

## Abstract

**Objectives:**

To evaluate the occurrence, risk factors, and outcomes of post PCNL (Percutaneous Nephrolithotomy) Acute Kidney Injury (AKI), with a secondary goal of developing a nomogram for post-PCNL AKI prediction.

**Methods:**

A prospective observational study was conducted enrolling 333 patients who underwent PCNL between February 2022 and February 2023. Patient demographics, comorbidities, perioperative lab parameters, stone characteristics, intraoperative details, and postoperative AKI were assessed. Logistic regression analyses were employed to construct a nomogram for predicting post-PCNL AKI.

**Results:**

40 patients (12.4%) experienced postoperative AKI, with recovery observed in all cases during the 3-month follow-up. Female gender (*p* = 0.002), hypertension(*p* = 0.022), higher serum uric acid levels(*p* = 0.003), staghorn calculi(*p* = 0.001), higher Hounsfield Units(*p* = 0.013), bilateral PCNL(*p* < 0.001), larger tract size(*p* = 0.017), longer operative time(*p* < 0.001), greater stone volume(*p* = 0.025), higher baseline serum creatinine levels(*p* < 0.001), higher postoperative total leukocyte count(*p* = 0.005), and postoperative fever(*p* < 0.001) were significantly associated with the AKI group. Regression analysis identified female gender (OR = 0.26, *p* = 0.035), higher serum uric acid levels(OR = 1.62, *p* = 0.013), bilateral PCNL(OR = 12.55, *p* < 0.001), longer operation time(OR = 1.02, *p* = 0.047), and larger stone volume(OR = 1.12, *p* = 0.015) as independent risk factors for postoperative AKI. The internally validated nomogram(*n* = 70) for predicting AKI demonstrated excellent diagnostic performance, with an area under the ROC curve of 0.984(95% CI, *p* < 0.001).

**Conclusion:**

AKI occurs in approximately 12% of patients undergoing PCNL. We identified several significant predictors of post-PCNL AKI, including female gender, hypertension, hyperuricemia, higher Hounsfield units, larger stone volume, bilateral PCNL, larger access tract size, and longer operative time. Awareness of these factors is crucial for optimizing management and improving patient outcomes.

**Supplementary Information:**

The online version contains supplementary material available at 10.1007/s00345-025-05511-w.

## Introduction

Kidney stones are a common health issue, with an estimated global prevalence of 1–15% [1]. Deterioration of renal function and progression to Chronic Kidney Disease (CKD) is seldom attributed to renal calculi alone, but evidence suggests that a single kidney stone episode can be associated with a small yet increased likelihood of adverse renal outcomes compared to non-stone formers [2]. PCNL is the preferred endourological approach for treating renal stones larger than 2 cm [3, 4]. Nevertheless, its invasive nature raises concerns regarding potential nephron loss and consequent impact on renal function [5]. The incidence of acute kidney injury (AKI) postoperatively in urology patients ranges between 6.7% and 38.2% [6]. However, prospective studies on its assessment are lacking. Several Nomograms have been developed to predict outcomes and stone free rates in the domain of urolithiasis, but there is a lack of nomograms focusing on the post operative renal function [7]. Our study aimed to assess the incidence, risk factors, and outcomes of post-PCNL AKI, with the secondary objective of constructing a nomogram for the prediction of post-PCNL AKI.

## Materials and methods

A prospective observational study was undertaken enrolling patients aged 18 years and older who underwent PCNL between February 2022 and February 2023. The study is registered with the Clinical Trials Registry (CTRI/2022/02/051922). The exclusion criteria included patients with preoperative AKI, those in the paediatric age group, pregnant women, those with active urinary tract infection and with bleeding diathesis. Based on prior studies suggesting an incidence of postoperative AKI of 16% in patients undergoing PCNL [6], and with a 95% confidence interval and Type I error: α = 0.05, the minimum required sample size was calculated to be 207 patients.

Preoperative factors recorded included patient demographics (age, sex, BMI), comorbidities (diabetes mellitus, hypertension), medication history (ACE inhibitors, beta blockers), and previous renal surgeries. The laboratory parameters included pre- and postoperative serum creatinine levels (at 12, 24, and 48 h), haemoglobin levels, total leukocyte counts, platelet counts, and serum uric acid levels. Stone characteristics assessed by Non-Contrast Computer Tomography (NCCT) included *stone burden (volume = L × W × D × π × 0.167) measured in cubic centimetres*, stone location, Hounsfield Units (HU), and laterality (unilateral/bilateral).

Intraoperative factors included the calyx punctured, tract size, number of access tracts, *operative time (time from puncture to exit)*, need for blood transfusion, intraoperative hypotension, bilateral PCNL, and use of a ureteral stent or nephrostomy tube post procedure.

Postoperative factors included complications graded using the modified Clavien–Dindo system, postprocedural blood parameters (total leucocyte count, haemoglobin, and serum creatinine at 12, 24, and 48 h), the need for haemodialysis and progression to Chronic Kidney Disease (CKD). All patients underwent a scheduled follow-up at 1 month post procedure. *Postoperative AKI in our study was defined based on the KDIGO criteria*,* which include any of the following: an increase in serum creatinine ≥ 0.3 mg/dL within 48 h*,* an increase in serum creatinine ≥ 1.5 times the baseline within the previous 7 days*,* or a urine output ≤ 0.5 mL/kg/h for at least 6 h* [8]. Patients with postoperative AKI were monitored with serial creatinine values for up to 3 months.

The PCNL procedure followed a standard protocol with patients positioned prone under general anaesthesia. Antibiotic prophylaxis involved intravenous administration of a 3rd generation cephalosporin at induction. A tract size ≥ 24Fr was categorized as standard PCNL, and a tract size ≤ 22Fr was categorized as mini PCNL. Prewarmed saline served as the irrigation fluid during the procedure. Energy sources used for fragmentation included pneumatic lithotripsy, Holmium: YAG laser or a Thulium Fiber laser lithotripsy. A double J ureteric stent was inserted postoperatively in all patients, and the operative time was measured from initial puncture to skin closure. Percutaneous nephrostomy tubes were placed when deemed necessary by the operating surgeon. *As part of our protocol*,* nephrotoxic drugs*,* including aminoglycosides and NSAIDs*,* were avoided in patients undergoing PCNL.*

Statistical analysis was performed using SPSS v23 (IBM Corp.). Descriptive statistics are presented as the means/standard deviations and medians/IQRs for continuous variables and as frequencies and percentages for categorical variables. Group comparisons for continuously distributed data were made using the independent sample t test when comparing two groups. The chi-squared test was used for group comparisons of categorical data. When the expected frequency in the contingency tables was < 5 for > 25% of the cells, Fisher’s exact test was used instead. Linear correlation between two continuous variables was explored using Pearson’s correlation (if the data were normally distributed) and Spearman’s correlation (for nonnormally distributed data). *p* < 0.05 indicated statistical significance.

## Results

A total of 333 patients were enrolled in the study, and 11 (3.3%) patients who were diagnosed with CKD preoperatively were excluded. Mean(SD) age of the study group was 48(13) years, with 68.9% being male. Mean(SD) BMI was 27.01 kg/m^2^. 76(23.6%) patients were diabetic and 95(29.5%) patients were hypertensive. Mean(SD) Stone volume was 3.13(3.69) cc, with 30(9.3%) patients having staghorn calculi. Mean(SD) stone density was 965(272) HU. 19(5.9%) patients underwent bilateral PCNL. 300(93.2%) patients had single access tract, while 22(6.8%) patients required multiple access tracts. Mean(SD) tract size was 24.78(5.25) Fr. Demographic details, stone characteristics, laboratory parameters and procedural details are summarized in supplementary Table 1 and supplementary Figure 1.

*Forty participants(12.4%) experienced postoperative AKI*,* as defined by the KDIGO criteria and all were followed up for 3 months postoperatively with no losses to follow-up.* During the 3-month follow-up, all 40 patients recovered from AKI. One patient did progress to AKI stage 3 postoperatively, requiring two sessions of renal replacement therapy; however, at the subsequent 3-month follow-up, the patient had recovered completely from AKI. There was no significant difference between the AKI and non-AKI groups in terms of age, BMI, or haemoglobin level. There was a significant difference in sex distribution(*p* = 0.002), hypertension(*p* = 0.022), and serum uric acid levels(*p* = 0.003), distributions of staghorn calculi(*p* = 0.001), Hounsfield Units(*p* = 0.013), bilateral PCNL(*p* < 0.001), tract size(*p* = 0.017), operative time(*p* < 0.001), stone volume(*p* = 0.025), baseline serum creatinine levels(*p* < 0.001) and postoperative total lymphocyte count(*p* = 0.005) between the AKI and non-AKI groups. The occurrence of postoperative fever was significantly greater in the AKI group(*p* < 0.001). The associations of various factors with AKI are summarized in supplementary Table 2.

Regression analysis(Table 1) revealed female sex(OR = 0.26, p = 0.035), higher serum uric acid levels(OR = 1.62, p = 0.013), B/L PCNL(OR = 12.55, p < 0.001), longer operation time (mean > 90 min)(OR = 1.02, p = 0.047), and larger stone volume (mean > 5 cc) (OR = 1.12, p = 0.015) as independent predictors of postoperative AKI.

**Table 1:** Regression analysis

Dependent: AKI		No	Yes	Odds Ratio (Univariate)	Odds Ratio (Multivariate)
Age (Years)	Mean (SD)	48.7 (12.9)	50.0 (15.4)	1.01 (0.98–1.03, *p* = 0.575)	1.00 (0.96–1.03, *p* = 0.838)
Gender	Male	186 (83.8)	36 (16.2)	-	-
Gender	Female	96 (96.0)	4 (4.0)	0.22 (0.06–0.56, *p* = 0.005)	0.26 (0.06–0.81, *p* = 0.035)
Diabetes Mellitus	Yes	62 (81.6)	14 (18.4)	1.91 (0.92–3.83, *p* = 0.073)	1.85 (0.69–4.88, *p* = 0.216)
Hypertension	Yes	77 (81.1)	18 (18.9)	2.18 (1.10–4.28, *p* = 0.024)	1.76 (0.65–4.83, *p* = 0.267)
Serum Uric Acid (mg/dL)	Mean (SD)	5.0 (1.1)	5.7 (1.4)	1.74 (1.29–2.41, *p* < 0.001)	1.62 (1.12–2.41, *p* = 0.013)
Staghorn Calculus	Yes	20 (66.7)	10 (33.3)	4.37 (1.81–10.05, *p* = 0.001)	2.87 (0.83–9.25, *p* = 0.083)
Hounsfield Units	Mean (SD)	952.8 (269.9)	1051.8 (280.7)	1.00 (1.00–1.00, *p* = 0.033)	1.00 (1.00–1.00, *p* = 0.299)
B/L PCNL	Yes	6 (31.6)	13 (68.4)	22.15 (8.09–67.54, *p* < 0.001)	12.55 (3.56–49.22, *p* < 0.001)
Track Size (Fr)	Mean (SD)	24.5 (5.3)	26.6 (4.4)	1.09 (1.02–1.18, *p* = 0.020)	1.08 (0.98–1.20, *p* = 0.149)
Operative Time	Mean (SD)	77.6 (21.0)	97.1 (26.2)	1.03 (1.02–1.05, *p* < 0.001)	1.02 (1.00-1.03, *p* = 0.047)
Volume of the Stone (cc)	Mean (SD)	2.8 (2.9)	5.5 (6.8)	1.15 (1.07–1.24, *p* < 0.001)	1.12 (1.02–1.24, *p* = 0.015)

MODEL FIT: χ²(11) = 83.95, p = < 0.001 Pseudo-R² = 0.35

Number in data frame = 322, Number in model = 322, Missing = 0

AIC = 181.7, C-statistic = 0.875, H&L = Chi-square(8) 6.51 (*p* = 0.591)

**Fig. 1:**
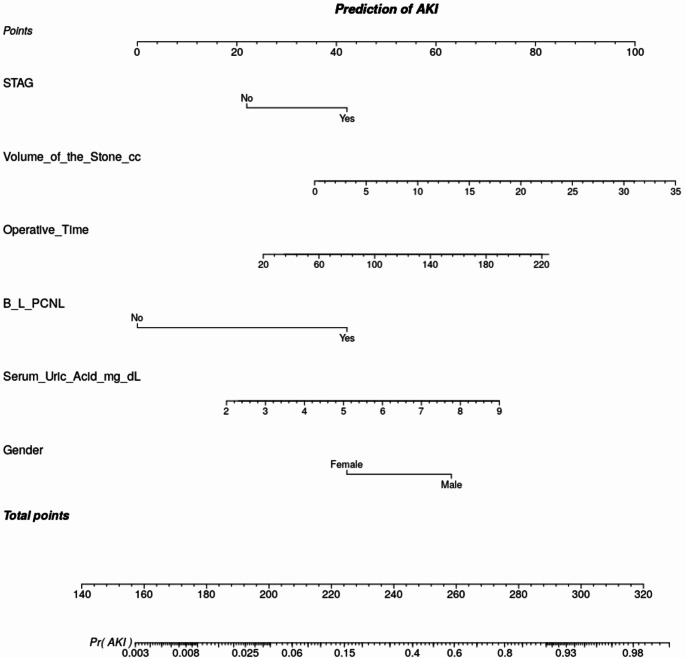
Nomogram predicting post-operative AKI

Based on these logistic regression analyses, a nomogram (Figure 1) was constructed that can predict post-PCNL AKI, with the points scale ranging from 140 to 320. Staghorn calculi were incorporated into the nomogram despite not demonstrating statistical significance in multivariate analysis. This decision was influenced by their substantial association in univariate analysis and their theoretical importance, as supported by prior studies [9]. The nomogram for predicting AKI underwent internal validation in a separate cohort of 70 patients not included in the study population and showed excellent diagnostic performance. The area under the ROC curve for the total score for predicting AKI was 0.984, which was statistically significant(*p* < 0.001). A cutoff total score ≥ 226 was used to predict AKI with a sensitivity of 100% and specificity of 97% (Supplementary Figure 3).

## Discussion

The impact of urolithiasis on renal function is complex and influenced by both the stone disease itself and the interventions employed. Although endourological procedures may not always affect global kidney function, certain factors, such as preoperative renal function, hypertension, diabetes mellitus, and the use of multiple percutaneous tracts, can predispose patients to declines in renal function following the procedure [10]. There exists a lack of epidemiological data on the incidence of AKI in the Indian population with urolithiasis in comparison to Non-Indian population.

Results of our study estimated the incidence of post-PCNL AKI to be 12.4%. This is in accordance with the finding of the previous studies, which estimate the range of incidence of post-PCNL AKI to be between 4.4–16.2% [6, 11]. This suggests that PCNL has a higher incidence of postoperative AKI when compared to other non-cardiac surgery, which have an incidence ranging from 0.8 to 7.5% [12, 13].

There was no statistically significant correlation between age or BMI and post-PCNL AKI, although older age [14] and higher BMI [15] are typically associated with a greater risk of AKI due to factors such as comorbidities and medication-induced renal stress, as well as age-related structural and functional changes, obesity-related glomerular hyperperfusion, oxidative stress and hyperfiltration injury. Although our study revealed a significant association between female sex and postoperative AKI, evidence supporting this finding is lacking. Further validation through larger sample sizes and randomized controlled trials is required to ensure its validity and reliability.

Findings from this study failed to demonstrate a significant correlation between post-PCNL AKI and diabetes, even though AKI is a known complication of uncontrolled diabetes mellitus [16] and is often associated with an increased risk of progression to chronic kidney disease and end-stage renal disease. It should be noted that PCNL is typically performed after achieving adequate diabetic control, which may explain the lack of significant correlation in our findings.

There was a significant correlation between hypertension and AKI post-PCNL in this study, highlighting the increased risk of AKI in hypertensive patients. This can be explained by Hypertension, similar to diabetes, being a systemic condition associated with progressive renal damage [17]. This creates a vicious cycle where hypertension exacerbates renal damage, which in turn worsens hypertension, and so on. Therefore, The importance of adequate blood pressure control in mitigating this risk cannot be overemphasized.

We did not find a significant correlation between preoperative Haemoglobin levels or total leukocyte count and post-PCNL AKI, despite Inflammation [18] and anaemia [19] being established factors that can contribute to AKI due to their capacity to cause renal hypoxia and oxidative stress. However, Notably, PCNL was performed after excluding active UTIs by negative urine cultures and the absence of clinical signs of pyelonephritis. Additionally, PCNL is rarely performed in anaemic patients before preoperative optimization, except in special circumstances such as CKD patients. This may explain the lack of correlation observed in our study.

Hyperuricemia was found to be a significant risk factor for post-PCNL AKI, highlighting the importance of the medical management of hyperuricemia in reducing this risk. Hyperuricemia is associated with a dysfunctional endothelial lining and heightened oxidative stress in the body, contributing to preglomerular arteriolopathy and the transmission of systemic hypertension to the glomerular capillary tuft. Even mild increases in serum uric acid levels can disrupt renal autoregulation and induce vasoconstriction, leading to a reduced glomerular filtration rate (GFR) and renal blood flow, thereby increasing the risk of AKI [20]. A study by Yu et al. similarly demonstrated higher preoperative serum uric acid levels as an independent risk factor for postoperative acute kidney injury (AKI), consistent with the findings of our study [21].

*Staghorn calculi*,* Hounsfield units (mean 1051 HU)*,* and stone volume (mean 5.5 cc) emerged as statistically significant predictors of post-PCNL AKI in this study.* These findings are in alignment with existent data on stone factors, including high-volume stones, staghorn stones, and higher Hounsfield units, contributing to procedural complexity, prolonged intraoperative time, and increased risks of bleeding and infective complications, thereby increasing the risk of AKI [22, 23].

Intraoperative factors, particularly increasing tract size, multiple access tracts, and bilateral PCNL were identified as significant predictors of post-PCNL AKI. Multiple access tracts, larger tract sizes, and bilateral PCNL procedures can result in significant renal parenchymal and nephron damage, leading to AKI, as supported by previous studies [22, 24–26]. While smaller tracts may predispose patients to greater intrarenal pressures and septic complications, we found that access tracts of 26 Fr or larger were significantly more strongly associated with post-PCNL AKI than smaller tracts were. Additionally, our observations revealed that out of 40 patients who developed AKI, 19 had undergone bilateral PCNL, emphasizing its negative impact on renal function.

*We observed that a mean operative time of (97 ± 26.24 min) was associated with an increased risk of postoperative AKI following PNL.* A prolonged operative time during PCNL procedures can increase the risk of complications such as haemorrhage and sepsis [27]. Longer procedures often indicate greater surgical complexity, potentially leading to additional insults to the kidney, particularly in the form of septic complications. However, factors such as calyx puncture, number of punctures, intraoperative hypotension, and blood transfusion did not show significant associations with post-PCNL AKI in our study.

Our study underscores the importance of addressing modifiable risk factors such as hypertension (HTN), hyperuricemia, tract size, operative time, and bilateral percutaneous nephrolithotomy (PNL) based on individual patient characteristics. Preoperative control of HTN, management of hyperuricemia, staged bilateral PNL procedures, and minimization of operative time are strategies that can potentially mitigate the risk of AKI following PNL. Furthermore, while certain risk factors, such as Female sex, staghorn calculi, Hounsfield units, and stone volume, are nonmodifiable, they remain crucial considerations in patient management. Endourologists should remain aware of these factors during the treatment of calculi disease to minimize the likelihood of post-PNL AKI.

Based on regression analysis, we developed a nomogram to predict post-PCNL AKI, which demonstrated good efficacy in a validation cohort of 70 patients. Nomograms that have been developed in urolithiasis have predominantly concentrated on the rates of stone clearance and postoperative complications as a whole, but our nomogram specifically targeting the predictive capacity of postoperative AKI in patients undergoing PCNL promises to be a novel tool that has not been extensively explored. However, larger validation studies are warranted to further assess its accuracy and reliability.

The strength of our study lies in its comprehensive characterization of the AKI in a large patient cohort undergoing PCNL, assessing multiple risk factors contributing to the development of AKI. Our study is limited by its single-center design, which restricts the generalizability of findings to other populations undergoing PNL for renal stones. Additionally, due to the short follow-up period of three months, the long-term outcomes of kidney function in patients with post-PNL AKI remain uncertain. A longer follow-up period, extended up to one year, would enable a more comprehensive assessment of overall outcomes and better predict renal function recovery in these patients. To enhance the reliability of our results, future studies could employ a randomized controlled design. *The lack of an accurate measurement of intraoperative blood loss*,* which could influence the development of postoperative AKI*,* is another limitation of our study.* Lastly, AKI was defined based on serum creatinine levels without considering hourly urine output, which is a component of AKI definition. Future studies should also include patient related quality of life and practical considerations in the use of nomograms and scoring systems in these patients [28, 29].

## Conclusion

In conclusion, our study identified several significant predictors of post-PNL AKI, including female sex, hypertension, hyperuricemia, staghorn calculi, higher Hounsfield units, larger stone volume, bilateral PNL, larger tract size, and longer operative time. Awareness of these factors is crucial for urologists to optimize management approaches and minimize the risk of post-PNL AKI, thereby improving patient outcomes. The nomogram developed in our study holds promise as a tool to aid clinicians in predicting post-PCNL AKI, although further validation is warranted to enhance its reliability and applicability in clinical practice.

## Electronic supplementary material

Below is the link to the electronic supplementary material.

Supplementary Material 1: Supplementary Table 1: Patient demographics, stone characteristics, and laboratory and procedural details. Supplementary Table 2: Association of perioperative parameters with AKI.

Supplementary Material 2: Supplementary Figure 1: Flow diagram depicting the methodology of the study

Supplementary Material 3: Supplementary Figure 2: ROC curve - the diagnostic performance of the probability of predicting AKI

## Data Availability

No datasets were generated or analysed during the current study.
