# Supplementary material for: “Development and validation towards a Nomogram to predict acute kidney Injury following PCNL”
Source: World J Urol. 2025 Feb 24;43(1):136. doi: 10.1007/s00345-025-05511-w (PMC11850472; doi:10.1007/s00345-025-05511-w)
Supplement: Supplementary file 1 — Supplementary Material 1: Supplementary Table 1: Patient demographics, stone characteristics, and laboratory and procedural details. Supplementary Table 2: Association of perioperative parameters with AKI. [file 345_2025_5511_MOESM1_ESM.docx]

Table 1: Patient demographics, stone characteristics, and laboratory and procedural details

| **Clinical Details** | **Mean ± SD \|\| Median (IQR) \|\| Min-Max OR N (%)** |  |
| --- | --- | --- |
| ***Demographics*** | |  |
| **Age (Years)** | 48.88 ± 13.18 \|\| 49.50 (38.25-57.75) \|\| 18.00 - 85.00 |  |
| **Male** | 222 (68.9%) |  |
| **Female** | 100 (31.1%) |  |
| **BMI (Kg/m²)** | 27.01 ± 3.05 \|\| 27.60 (25.00-29.00) \|\| 19.80 - 32.90 |  |
| **DM (Yes)** | 76 (23.6%) |  |
| **HTN (Yes)** | 95 (29.5%) |  |
| **Anomaly (Yes)** | 6 (1.9%) |  |
| **ACE Inhibitors (Yes)** | 0 (0.0%) |  |
| **Beta Blockers (Yes)** | 19 (5.9%) |  |
| **Previous Renal Surgery (Yes)** | 21 (6.5%) |  |
| ***Stone Characteristics*** | |  |
| **Hounsfield Units** | 965.06 ± 272.75 |  |
| **Stone Volume** **(cc)** | 3.13 ± 3.69 \|\| 1.98 (0.92-3.76) \|\| 0.36 - 31.08 |  |
| **Staghorn calculi (Yes)** | 30 (9.3%) |  |
| ***Laboratory Parameters*** | |  |
| **Haemoglobin (g/dL)** | 13.12 ± 1.90 |  |
| **Total count (x10³/mm³) (Baseline)** | 8.23 ± 2.19 |  |
| **Platelet Count (x10³/mm³)** | 276.31 ± 92.19 |  |
| **Serum Uric Acid (mg/dL)** | 5.09 ± 1.20 |  |
| ***Procedural Details*** | |  |
| **B/L PNL (Yes)** | 19 (5.9%) | |
| **Superior Calyceal Access** | 38 (11.8%) | |
| **Middle Calyceal Access** | 65 (20.2%) | |
| **Inferior Calyceal Access** | 219 (68.0%) | |
| **Supracostal Puncture** | 48 (14.9%) | |
| **Infracostal Puncture** | 274 (85.1%) | |
| **Single Access Tract** | 300 (93.2%) | |
| **Multiple Access Tracts** | 22 (6.8%) | |
| **Track Size (Fr)** | 24.78 ± 5.25 \|\| 27.00 (21.00-30.00) \|\| 15.00 - 34.00 | |
| **Blood Transfusion (Yes)** | 3 (0.9%) | |
| **Operative Time** | 80.04 ± 22.57 \|\| 75.00 (65.00-90.00) \|\| 35.00 - 225.00 | |
| **Intra-Operative Hypotension (Yes)** | 3 (0.9%) | |

Table 2: Association of perioperative parameters with AKI

| **Parameters** | **AKI** | | **p value** |
| --- | --- | --- | --- |
|  | **Yes (n = 40)** | **No (n = 282)** |  |
| **Age (Years)** | 49.98 ± 15.36 | 48.73 ± 12.86 | 0.626^1^ |
| **Gender***** |  |  | 0.002^3^ |
| Male | 36 (90.0%) | 186 (66.0%) |  |
| Female | 4 (10.0%) | 96 (34.0%) |  |
| **BMI (Kg/m²)** | 27.64 ± 3.00 | 26.93 ± 3.05 | 0.166^4^ |
| **DM (Yes)** | 14 (35.0%) | 62 (22.0%) | 0.070^3^ |
| **HTN (Yes)***** | 18 (45.0%) | 77 (27.3%) | 0.022^3^ |
| **Anomaly (Yes)** | 2 (5.0%) | 4 (1.4%) | 0.164^2^ |
| **ACE Inhibitors (Yes)** | 0 (0.0%) | 0 (0.0%) | 1.000^3^ |
| **Beta Blockers (Yes)** | 2 (5.0%) | 17 (6.0%) | 1.000^2^ |
| **Previous Renal Surgery (Yes)** | 4 (10.0%) | 17 (6.0%) | 0.312^2^ |
| **Hemoglobin (g/dL)** | 13.02 ± 1.91 | 13.13 ± 1.90 | 0.999^4^ |
| **TLC (x10³/mm³) (Baseline)** | 7.57 ± 1.93 | 8.33 ± 2.21 | 0.100^4^ |
| **Platelet Count (x10³/mm³)** | 283.30 ± 93.29 | 275.31 ± 92.16 | 0.609^4^ |
| **Serum Uric Acid (mg/dL)***** | 5.73 ± 1.43 | 5.00 ± 1.14 | 0.003^1^ |
| **Staghorn Calculus (Yes)***** | 10 (25.0%) | 20 (7.1%) | 0.001^2^ |
| **Hounsfield’s Units***** | 1051.75 ± 280.70 | 952.77 ± 269.86 | 0.013^4^ |
| **B/L PCNL (Yes)***** | 13 (32.5%) | 6 (2.1%) | <0.001^2^ |
| **Calyx Punctured** |  |  | 0.697^3^ |
| Superior | 6 (15.0%) | 32 (11.3%) |  |
| Middle | 9 (22.5%) | 56 (19.9%) |  |
| Inferior | 25 (62.5%) | 194 (68.8%) |  |
| **Incision Site** |  |  | 0.055^3^ |
| Supracostal | 10 (25.0%) | 38 (13.5%) |  |
| Infracostal | 30 (75.0%) | 244 (86.5%) |  |
| **Number of Punctures** |  |  | 0.744^2^ |
| Single | 37 (92.5%) | 263 (93.3%) |  |
| Multiple | 3 (7.5%) | 19 (6.7%) |  |
| **Track Size (Fr)***** | 26.62 ± 4.36 | 24.52 ± 5.32 | 0.017^4^ |
| **Blood Transfusion (Yes)** | 0 (0.0%) | 3 (1.1%) | 1.000^2^ |
| **Operative Time***** | 97.12 ± 26.24 | 77.62 ± 20.95 | <0.001^4^ |
| **Volume of the Stone (cc)***** | 5.53 ± 6.82 | 2.79 ± 2.86 | 0.025^4^ |
| **Intra-Operative Hypotension (Yes)** | 1 (2.5%) | 2 (0.7%) | 0.329^2^ |
| **S. Creatinine (Baseline)***** | 1.16 ± 0.35 | 0.97 ± 0.43 | <0.001^4^ |
| **Postoperative Fever (Yes)***** | 9 (22.5%) | 13 (4.6%) | <0.001 |
| **TLC (x103/mm3) (Post-Op) ***** | 12.77 ± 5.15 | 10.47 ± 3.93 | 0.005^4^ |
| p values are derived from Mann–Whitney U test or T test for continuous variables, and Chi-square test for categorical variables Bold values indicate p ≤ 0.05 | | | |
