# Supplementary figures and images for: “Development and validation towards a Nomogram to predict acute kidney Injury following PCNL”
Source: World J Urol. 2025 Feb 24;43(1):136. doi: 10.1007/s00345-025-05511-w (PMC11850472; doi:10.1007/s00345-025-05511-w)

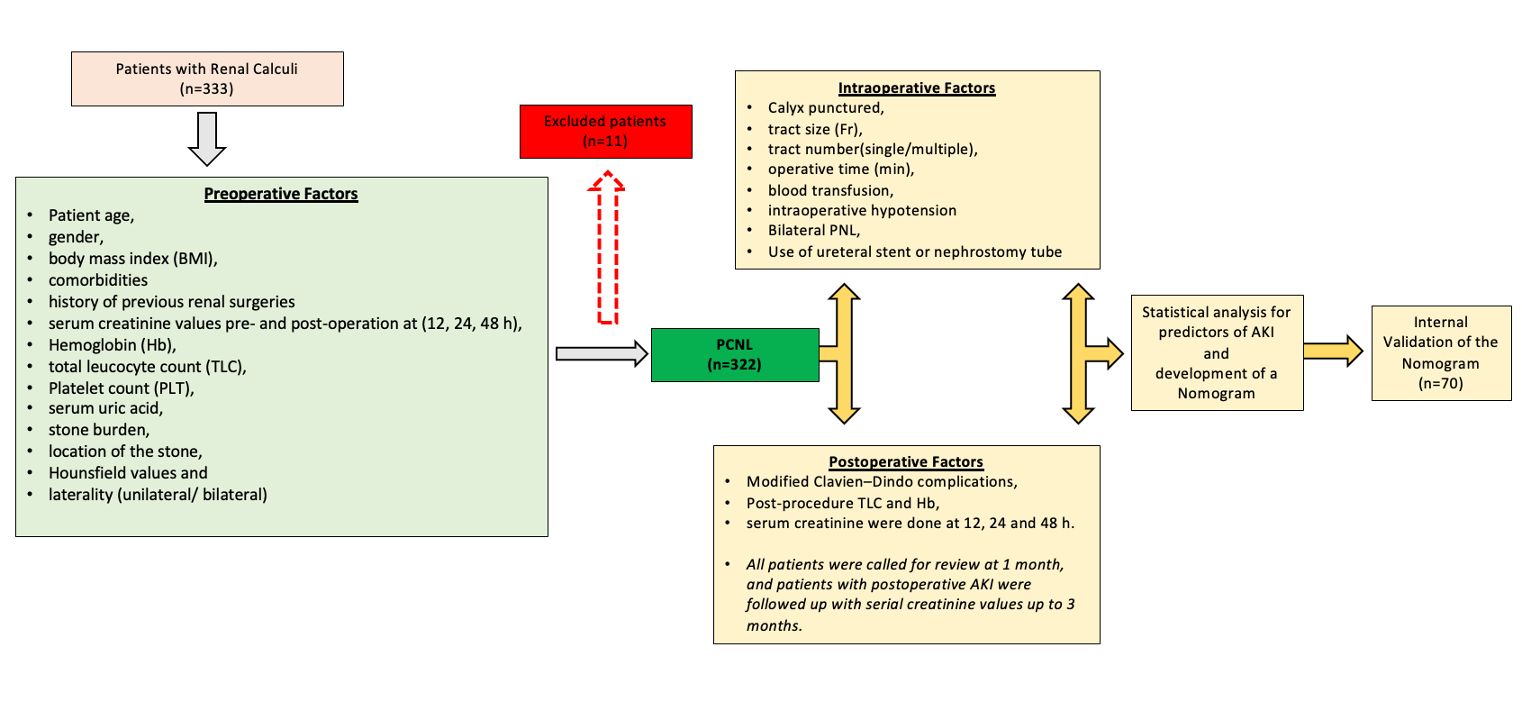

Supplement: Supplementary file 2 — Supplementary Material 2: Supplementary Figure 1: Flow diagram depicting the methodology of the study [file 345_2025_5511_MOESM2_ESM.png]

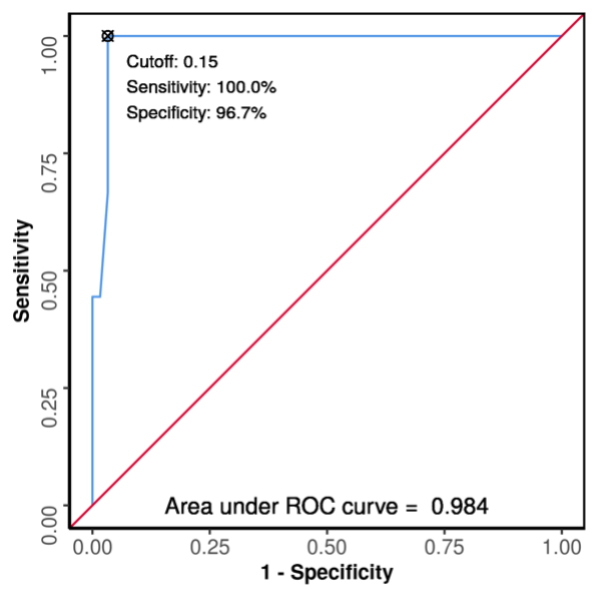

Supplement: Supplementary file 3 — Supplementary Material 3: Supplementary Figure 2: ROC curve - the diagnostic performance of the probability of predicting AKI [file 345_2025_5511_MOESM3_ESM.png]
